# Immunofluorescence microscopy-based detection of ssDNA foci by BrdU in mammalian cells

**DOI:** 10.1016/j.xpro.2021.100978

**Published:** 2021-11-25

**Authors:** Susan Kilgas, Anne E. Kiltie, Kristijan Ramadan

**Affiliations:** 1 MRC Oxford Institute for Radiation Oncology, Department of Oncology, University of Oxford, Oxford OX3 7DQ, UK; 2 Rowett Institute, University of Aberdeen, Aberdeen AB25 2ZD, UK

**Keywords:** Cell Biology, Cell-based Assays, Microscopy, Molecular Biology, Antibody

## Abstract

DNA end resection converts broken ends of double-stranded DNA (dsDNA) to 3′-single-stranded DNA (3′-ssDNA). The extent of resection regulates DNA double-strand break (DSB) repair pathway choice and thereby genomic stability. Here, we characterize an optimized immunofluorescence (IF) microscopy-based protocol for measuring ssDNA in mammalian cells by labeling genomic DNA with 5-bromo-2′-deoxyuridine (BrdU). BrdU foci can be detected under non-denaturing conditions by anti-BrdU antibody, providing an accurate and reliable readout of DNA end resection in most mammalian cell lines.

For complete details on the use and execution of this protocol, please refer to Kilgas et al. (2021).

## Highlights

• Single-stranded DNA (ssDNA) detection by BrdU labeling under non-denaturing conditions
• Number of ssDNA foci measured by immunofluorescence (IF)-based confocal microscopy
• Compatible with co-staining for cell cycle markers
• Amenable to semi-quantitative image analysis for quantification

## Graphical abstract

**Figure:**
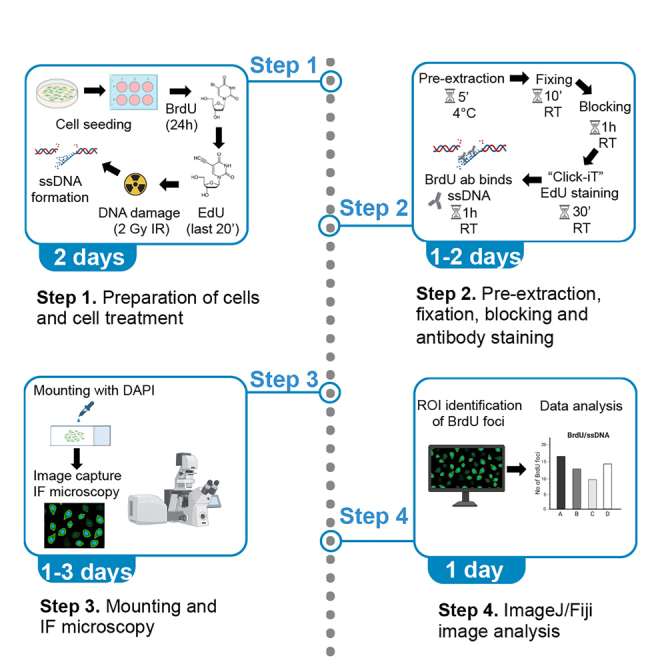

## Before you begin

The protocol below describes the detailed steps for immunofluorescence microscopy (IF)-based detection and quantification of ssDNA foci upon BrdU labeling under native (non-denaturing) conditions. Cells are optionally exposed to ionising irradiation (IR) or hydroxyurea (HU) as a strategy to induce DNA damage/DSB and consequently initiate DNA end resection. This protocol is optimized for T24 human urinary bladder cancer cells, although variations of this protocol exist in several mammalian cell lines subjected to various DNA damaging agents *in vitro* (He et al., 2018; Broderick et al., 2016; Kao et al., 2001; Halder et al., 2019). Specifically, this protocol: (1) offers a simple cell culture-based technique to label ssDNA in cells exposed to DNA damage; (2) quantifies the number of ssDNA foci per cell using a semi-quantitative method; (3) involves simultaneous labeling of cell cycle markers to study any cell-cycle specific effects on DNA end resection.

## Key resources table

**Table:** 

REAGENT or RESOURCE	SOURCE	IDENTIFIER
**Antibodies**		

5-Bromo-2′-deoxyuridine (BrdU) Mouse monoclonal (concentration: 1:300)	BD Biosciences	Cat# 347580; RRID:AB_10015219
Cyclin A2 antibody (concentration: 1:500)	Novus Biologicals	Cat# NBP1-31330, RRID:AB_10003781
Alexa Fluor 488 Mouse polyclonal (concentration: 1:500)	Thermo Fisher Scientific	Cat# A-21202; RRID:AB_141607
Alexa Fluor 568 Rabbit polyclonal (concentration: 1:500)	Thermo Fisher Scientific	Cat#A-11011; RRID: AB_143157

**Chemicals, peptides, and recombinant proteins**		

BrdU	Sigma-Aldrich	Cat# B5002
5-Ethynyl-2′-deoxyuridine (EdU)	Thermo Fisher Scientific	Cat# A10044
Dimethyl sulfoxide (DMSO)	Sigma-Aldrich	Cat# D2650
Sodium azide 0.1 M solution	Sigma-Aldrich	Cat# 08591
ProLong Diamond antifade with DAPI	Thermo Fisher Scientific	Cat# P36962
Hoechst 33342	Sigma-Aldrich	Cat# B2261
4′-6-Diamidino-2-Phenylindole, Dihydrocloride (DAPI)	Thermo Fisher Scientific	Cat #D1306
Immuno-Mount Aqueous Non-fluorescing Mounting Medium	Interchim	Cat# 983260
Bovine Serum Albumin (BSA)	Sigma-Aldrich	Cat# 9048-46-8
Fetal Bovine Serum (FBS)	Thermo Fisher Scientific	Cat# 11573397
Trypsin-EDTA (0.25%)	Thermo Fisher Scientific	Cat# 25200072
Phosphate buffered saline (PBS)	Gibco	Cat# 10010023
Hydroxyurea (HU)	Sigma	Cat# H8627
Triton X-100	Sigma-Aldrich	Cat# T8787
4-(2-hydroxyethyl)-1-piperazineethanesulfonic acid (HEPES), pH 7.5	Sigma-Aldrich	Cat# H4034
Sucrose	Sigma-Aldrich	Cat# S0389
Magnesium chloride (MgCl_2_)	Thermo Fisher Scientific	Cat# AM9530G
Sodium chloride (NaCl)	Sigma-Aldrich	Cat# S5150
Ethylenediaminetetraacetic acid (EDTA)	Thermo Fisher Scientific	Cat# 15575020
Paraformaldehyde (PFA) 36% (39% w/v)	VWR	Cat# 50-00-0
Click-IT EdU Alexa Fluor 555 imaging kit	Thermo Fisher Scientific	Cat# C10338
McCoy’s 5A Medium	Thermo Fisher Scientific	Cat# 16600082
Trypan blue solution	Thermo Fisher Scientific	Cat#15250061

**Experimental models: Cell lines**		

Human: T-24 urinary bladder cancer, transitional cell carcinoma grade III	German Collection of Microorganisms and Cell Cultures (DSMZ)	DSMZ No 376; RRID:CVCL_0554

**Software and algorithms**		

Prism 8	GraphPad Software	https://www.graphpad.com; RRID:SCR_002798
Fiji	NIH	https://imagej.net/Fiji/Downloads; RRID:SCR_002285

**Other**		

Round cover glasses; 9 mm; thickness: No. 1	VWR	Cat# 631-0169
6-well plate, round	Thermo Fisher Scientific	Cat# 174901
T75 flasks	Thermo Fisher Scientific	Cat# 156499
Forceps	Sigma-Aldrich	Cat# F3767
Kimwipes	Thermo Fisher Scientific	Cat# 06-666
Superfrost Plus Microscope Slides	Thermo Fisher Scientific	Cat #22-037-246
Caesium-137 GSR D1 gamma irradiator (1.5 Gy/min)	Gamma-Service Medical GmbH	N/A
Zeiss 710 confocal microscope	ZEISS	N/A
Nikon Ni-E epifluorescent microscope	Nikon	N/A

## Materials and equipment

**Table:** Preparation of BrdU stock solution

Compound	Final concentration	Amount
BrdU	10 μM	3.071 g
DMSO	0.1%	1 mL
**Total**	n/a	1 mL stock solution

***Note:*** Final concentration of 10 μM BrdU was obtained from a 10 mM stock by dissolving 3.071 g of BrdU powder (MW= 307.10 g/mol) in 1 mL DMSO.

**Table:** Preparation of EdU stock solution

Compound	Final concentration	Amount
EdU	10 μM	2.5223 g
DMSO	0.1%	1 mL
**Total**	n/a	1 mL stock solution

***Note:*** Final concentration of 10 μM EdU was obtained from a 10 mM stock by dissolving 3.071 g of BrdU powder (MW= 252.23 g/mol) in 1 mL DMSO.

**Table:** Fixing solution (prepare fresh, cool on ice before use)

Compound	Final concentration	Amount
Paraformaldehyde (PFA) (36%)	4%	5.55 mL
1× PBS	n/a	44.45 mL
**Total**	n/a	50 mL

**CRITICAL:** Paraformaldehyde (PFA) is classified as a hazardous chemical by the 2012 OSHA Hazard Communication Standard (29 CFR 1910.1200). Harmful if swallowed or inhaled (H302+H332). Causes skin irritation (H315), serious eye damage (H318). May cause respiratory irritation (H335). Suspected carcinogen (H351). Avoid contact with eyes, skin, or on clothing. Avoid ingestion and inhalation. Ensure adequate ventilation in the room of handling. Keep away from sources of ignition and heat. Wear personal protective equipment (PPE) when handling.

***Note:*** Paraformaldehyde (4%) fixing solution should be prepared fresh on the day.

**Table:** Blocking buffer

Compound	Final concentration	Amount
Bovine serum albumin (BSA)	5%	5 g
MilliQ water	n/a	95 mL
**Total**	n/a	100 mL

**CRITICAL:** Bovine serum albumin (BSA) is harmful if swallowed (H302). May be irritating to the mucous membranes and upper respiratory tract. May be harmful by inhalation or absorption by the skin, and cause eye irritation. Wear Personal Protective Equipment (PPE) when handling.

***Note:*** Blocking buffer can be maintained for weeks at 4°C upon addition of 1 mM sodium azide.

**Table:** Antibody dilution buffer

Compound	Final concentration	Amount
5% BSA in PBS	2.5%	5 mL
1× PBS	1:1 ratio with 5% BSA/PBS	5 mL
**Total**	n/a	10 mL

***Note:*** The volume of actual antibody dilution buffer depends on the number of samples and experiments. Recommended to use fresh immediately after preparation. Keep on ice or at 4°C.

**Table:** Pre-extraction buffer (prepare fresh, cool on ice before use)

Compound	Final concentration	Amount
Hepes pH 7.5 (1 M)	25 mM	250 μL
NaCl (5 M)	50 mM	100 μL
EDTA (500 mM)	1 mM	20 μL
MgCl_2_ (1 M)	3 mM	30 μL
Sucrose	300 mM	1.026 g
Triton X-100 (10% in ddH_2_O)	0.5%	0.5 mL
ddH_2_O	n/a	9.1 mL
**Total**	**n/a**	**10 mL**

**CRITICAL:** Triton X-100 is harmful if swallowed (H302). May cause serious eye damage (H318) and skin irritation (H315). Wear PPE when handling.

**CRITICAL:** Ethylenediaminetetraacetic acid (EDTA) is harmful if inhaled (H332). Avoid contact with eyes, skin, or clothing. Avoid inhalation and wear PPE when handling.

***Note:*** Pre-extraction buffer stock solutions can be stored at RT (20°C–22°C) for at least 1 month.

***Alternatives:*** Alternative cytoskeletal (CSK) pre-extraction buffer (Britton et al., 2013):

**Table:** 

Compound	Final concentration	Amount
PIPES pH 7.5 (0.5 M)	10 mM	200 μL
NaCl (5 M)	100 mM	200 μL
MgCl_2_ (1 M)	3 mM	100 μL
Sucrose	300 mM	1 g
Triton X-100 (10% in ddH_2_O)	0.7%	700 μL
ddH_2_O	n/a	8.8 mL
**Total**	**n/a**	**10 mL**

***Note:*** Pre-extraction buffer stock solutions can be stored at RT (20°C–22°C) for at least 1 month. PIPES should be stored at −20°C.

## Step-by-step method details

### Cell culture and BrdU treatment (± IR)

**Timing: 2 days**

1. Detach T24 cells from maintenance culture flasks (T75 flasks) using Trypsin-EDTA (0.25%) by incubating flasks for a few minutes at 37°C and 5% CO_2_.

**CRITICAL:** Cell culture should be performed under a Class II Biological Safety Cabinet. Pre-warm cell culture medium and Trypsin-EDTA solution to avoid excessive thermal shifts in cells; Before starting any treatments, check whether cell morphology appears normal, and make sure there is no contamination present in any of the wells. To avoid contamination, make sure the forceps and the coverslips are sterilized before using them for cell culture. Do not open cell culture forceps outside the biological safety cabinet and always sterilize with 70% ethanol after use. Coverslip sterilization can be achieved by autoclaving.

***Note:*** Cell detachment time depends on the cell type and the presence of medium leftovers in the flask. Before proceeding with next steps, ensure that cells are detached from culture flasks.

***Alternatives:*** Mechanical scraping using sterile scrapers and PBS or PBS-EDTA solution (3 mM EDTA) can be used instead of trypsin-EDTA.

2. Neutralize trypsin by adding at least 2 volumes of complete media (McCoy’s 5A Medium with 10% FBS).
3. Collect cells into a Falcon tube of appropriate size.
4. Centrifuge cells at 900–1,000 × *g* at 20°C–22°C for 3–4 min.
5. Gently aspirate media so as not to disturb the cell pellet at the bottom.
6. Resuspend cell pellets in complete culture medium.
7. Seed cells into 6-well plates so they are around 50%–60% confluent the next day. Typically, for T24 cells: 0.4 × 10^6^ cells per well.

***Note:*** Each well in a 6-well plate is a separate condition, so the number of coverslips needed per well depends on how many time points and technical replicates are required for a specific experiment

8. Count cells in trypan blue solution (1:1 ratio of cells and trypan blue reagent) using a standard or automated hemocytometer.
9. Add sterilized round cover glasses (9 mm; thickness No.1) to the bottom of the wells with fresh media in it. Gently press down with forceps to make sure that the cover glasses stick to the bottom of the wells. Then, add cell suspension to the wells (total volume to 2 mL).
10. The next day, add 10 μM BrdU to 50%–60% confluent cells and keep on for 24 h.

***Note:*** When using other chemicals/inhibitors to study their effects on ssDNA generation, add these to the media with BrdU in the last few hours of the 24-h BrdU treatment at a concentration needed according to your specific compound. For example, add HU (500 μM) for 1 h before sample processing.

11. Add 10 μM EdU to cells recovered with the culture media containing BrdU in the last 20 min prior to cell fixation to mark cells in S phase.

***Alternatives:*** Instead of using EdU and the associated “Click-IT” reaction kit for S-phase staining outlined later in this protocol (step 20), cells can be stained with Cyclin A antibody instead. The staining procedure will be outlined below in step 35.

12. Remove media with BrdU/EdU after 20-min EdU treatment, treat cells with DNA damage inducing agents, recover over time or fix immediately.

**CRITICAL:** The recovery time between radiation/other DNA damaging agents and fixation may influence the amount of ssDNA detectable under the microscope (Kilgas et al., 2021). Radiation doses over 2–4 Gy can lead to exhaustion of the ssDNA-binding protein RPA involved in end resection (Toledo et al., 2013), and therefore we recommend limiting irradiation dose to no more than 4 Gy for ssDNA microscopy-based assays. Doses as high as 10 Gy will also result in more cell death and consequently fewer cells available for analysis.

13. Irradiate cells with 2 Gy IR. Non-irradiated cells can be kept in a separate 6-well plate and used as an internal control. During the recovery period after IR, control and irradiated cells are maintained in an incubator in complete media at 37°C and 5% CO_2_ for the desired amount of time prior to fixing and processing for IF.

***Note:*** Irradiation time to achieve 2 Gy total dose depends on the specific dose-rate used and varies between different irradiators.

***Alternatives:*** Both gamma- and X-ray based irradiators can be used. Alternatively, radiomimetic drugs such as bleomycin or neocarzinostatin can be used.

### Pre-extraction and cell fixation

**Timing: Approximately 30 min**

14. Transfer coverslips into individual wells in a 12-well plate and wash the media off with 1 mL 1× ice-cold PBS.
15. Aspirate PBS and add 1 mL of ice-cold pre-extraction buffer for 5 min over ice.

**CRITICAL:** Cells can become more likely to detach after pre-extraction. Handle coverslips gently and carefully after this step. Add all the buffers from the wall of the culture dish and not directly onto cells to avoid cell detachment. Also, make sure to keep coverslips on ice during pre-extraction, and do not exceed 5 min to preserve cells (see troubleshooting 3). Prepare a pre-extraction buffer fresh every time.

16. Aspirate pre-extraction buffer and wash once with 1 mL cold 1× PBS to remove pre-extraction buffer.
17. Add 1 mL of freshly prepared ice-cold 4% paraformaldehyde (PFA) in PBS to each well. Incubate for 10 min at 20°C–22°C.

**CRITICAL:** Prepare 4% PFA fixing solution fresh every time (see troubleshooting 1 and 2).

18. Wash cells twice with 1 mL 1× PBS at 20°C–22°C for 2–3 min each.

### Blocking and EdU detection for S-phase cell detection

**Timing: Approximately 1 h and 30 min**

19. Add 1 mL of 5% BSA in PBS and incubate for 1 h at 20°C–22°C
20. Prepare Click-iT reaction mixture according to the manufacturer’s protocol (Thermo Fisher).
21. Add 30 μL droplets of Click-iT reaction solution on a parafilm in a wet chamber.
22. Lift coverslips from the 12-well plate with forceps, dry extra liquid on a tissue paper and place upside down on the Click-iT reaction buffer.
23. Incubate for 30 min in the dark.
24. Transfer coverslips back to the 12-well plate before washing the cells.
25. Wash cells twice for 2–3 min.

**CRITICAL:** Make sure to keep coverslips in dark after the Click-IT reaction step, as the fluorescent azide dye is light-sensitive. When washing coverslips in the 12-well plate, cover the plates with aluminum foil during washes.

**Pause point:** Coverslips can be kept in the blocking solution at 4°C overnight (approximately 16 h) or up to a few days.

***Alternatives:*** Thermo Fisher Scientific **“**Click-iT” reaction kits come with a variety of different wavelengths for the fluorescent dye and can be chosen according to the choice of BrdU channel and DAPI channel, to avoid any potential cross-reactivity. Alternatively, “Click-IT” reaction kit can be replaced by staining cells with Cyclin A antibody, which would skip both the EdU labelling step (step 11) as well as the “Click-iT” step outlined in steps 20–25.

### Antibody staining

**Timing: 2 h and 30 min (up to 4 h and 30 min when Cyclin A antibody is added instead of EdU labelling)**

26. Dilute primary BrdU antibody in 2.5% BSA/PBS solution (BrdU antibody concentration: 1:300).

**CRITICAL:** Ensure the coverslip is fully covered in antibody solution (see troubleshooting 4).

27. Add 30 μL droplets of antibody solution on parafilm in a wet chamber.
28. Lift coverslips from the 12-well plate with forceps, dry extra liquid on a tissue paper and place upside down on the antibody droplet. Incubate for 1 h at 20°C–22°C in the dark.
29. Transfer the coverslips back to the 12-well plate, fixed cells facing up, and add 1 mL 1× PBS at 20°C–22°C into each well for 5 min, then gently remove PBS by using a vacuum pump. Repeat this step 2 additional times (washing step; a total of 3 washing steps).
30. Dilute secondary fluorescent antibody (Alexa Fluor 488) in antibody dilution buffer.
31. Add 30 μL droplets of antibody solution on parafilm in a wet chamber.
32. Lift coverslips from the 12-well plate using forceps as in (3), dry extra liquid and place coverslip upside down on antibody droplets.
33. Incubate for 1 h at 20°C–22°C in the dark.
34. Transfer the coverslips back to 12-well plate as in (4), and wash with 1 mL 1× PBS at 20°C–22°C for 3 times and 5 min each.
35. Dilute Cyclin A antibody in 2.5% BSA/PBS solution (Cyclin A antibody concentration: 1:500).
36. Repeat steps 27–29.
37. Dilute secondary fluorescent antibody (Alexa Fluor 594) in antibody dilution buffer.
38. Repeat steps 31–34.

**Pause point:** Coverslips can be kept in primary antibody dilution at 4°C overnight (approximately 16 h) provided they are in a wet chamber so that the antibody solution will not dry out.

### Mounting and microscopy image acquisition

**Timing: Approximately 1–3 days**

39. Use ProLong Diamond antifade with DAPI mounting solution to make individual droplets on microscopy slides and mount the coverslips (one droplet for each coverslip).

**CRITICAL:** Avoid bubbles when putting the mounting solution on microscopy slides as this will greatly decrease the quality of your imaging. Also, the mounting solution hardens relatively quickly, so make sure to add the mounting droplets just before adding coverslips on them (see troubleshooting 5).

***Alternatives:*** Instead of using the Prolong Diamond antifade DAPI mounting solution, cells can be incubated in DAPI (Thermo Fisher Scientific; Cat #D1306) with PBS (1 μg/mL final concentration) for 10 min in the dark and mounted using ProLong Glass Antifade Mountant (Thermo Fisher Scientific; Cat# P36980). After incubation with DAPI, wash cells once for 5 min with 1× PBS (Step 30); As an alternative to DAPI dye, nuclear staining can be done with Hoechst 33342 dye (Sigma-Aldrich; Cat# B2261) in PBS (5 μg/mL final concentration) for 10 min at 20°C–22°C in the dark, followed by mounting with ProLong Glass Antifade Mountant (Thermo Fisher Scientific; Cat# P36980).

40. Dip coverslips gently in ddH_2_O to remove salts.
41. Dry extra liquid on kimwipes.

**CRITICAL:** Drying extra liquid should be done superficially and quickly to avoid excessive dilution of the mounting medium. When allowed to completely dry, auto-fluorescence can interfere with sample staining (see troubleshooting 5).

42. Place coverslips sample-upper side down (fixed cells facing the mounting solution).
43. Incubate at 20°C–22°C in the dark for 30 min minimum (up to 24 h at 20°C–22°C to let the mounting medium cure).
44. Store at 4°C in the dark.

**Pause point:** Microscopy slides mounted with ProLong Diamond antifade with DAPI mounting solution can be kept at 4°C in the dark (to avoid photobleaching) for at least a month without losing quality of the images. However, it is recommended to image the slides as soon as possible.

45. Capture images using an epifluorescent or confocal microscope using the Use DAPI channel with a 10× lens to locate cells. Then change the magnification to either 40× or 63× oil lens, focus and capture images with DAPI channel (excitation: 359 nm), FITC channel (BrdU antibody; excitation: 488 nm) and TRITC channel (EdU label/Cyclin A antibody; excitation: 555 nm).

**CRITICAL:** Adjust laser power and gain without overexposing samples even in the brightest condition (see troubleshooting 6 and 7). Keep the same settings for each biological condition.

***Optional:*** The excitation wavelength can be different for BrdU and Edu-labelled cells, depending on which fluorescent antibody and dye you are using. There are several “Click” reaction kits with different wavelength dyes, and BrdU can be alternatively imaged at higher excitation wavelengths.

46. Save files as original multichannel/composite images because they contain the original pixels from the microscope. RGB images are good for display purposes as they have consistent appearance but have lost original pixel values, so are not suitable for accurate analysis.

**CRITICAL:** Multichannel/composite images may not be readable in some software but can be opened in imageJ/Fiji.

## Expected outcomes

**Figure 1:**
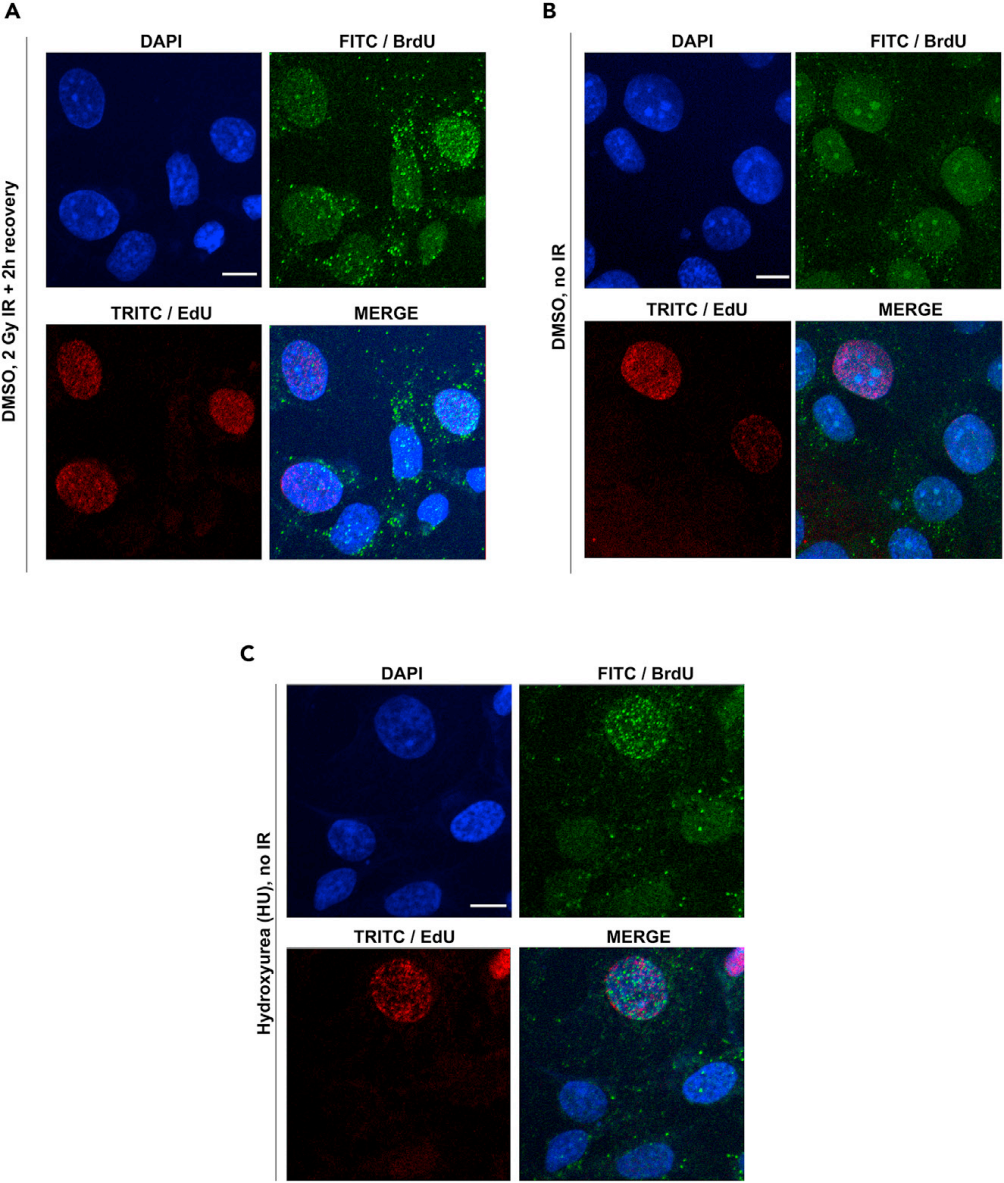
Confocal microscopy-based representative images showing BrdU foci during and outside S-phase (A and B) T24 cells treated with 2 Gy IR and recovered over 2 h show generation of nuclear BrdU/ssDNA foci. (B) DMSO-treated control (no IR) T24 cells showing no discernible nuclear BrdU/ssDNA foci (FITC/green channel) being formed either during or outside S-phase (TRITC/red channel). (C) HU-treated (500 μM for 1 h) positive control T24 cells showing representative formation of BrdU/ssDNA foci in S-phase cells only. Scale bar = 10 μm.

Confocal images of T24 cells exposed to 24-h BrdU treatment and 2 Gy IR show that cells stain positive for BrdU foci/ssDNA foci (Figure 1A). Because stimulating DNA end resection above background threshold and subsequent generation of long stretches of ssDNA requires DSB formation (Zhao et al., 2020), non-treated control cells have no noticeable BrdU foci (Figure 1B). When compared with IR-treated conditions, the DMSO control and HU-treated cells have minimal cytoplasmic staining of BrdU foci compared to IR-treated cells. One of the potential reasons for this could be that IR itself causes ssDNA accumulation in the cytoplasm (Ragu et al., 2020), and the residual cytoplasm that remains after pre-extraction will therefore stain for some ssDNA foci outside the nucleus. Additionally, there could be background foci present depending on the endogenous levels of DNA damage in specific cell lines, but compared to irradiated and treated conditions, they should be significantly less (Kilgas et al., 2021; Mukherjee et al., 2015). A positive control should be used when comparing between treatments, which here is shown as HU in S-phase cells (Feng et al., 2006; Sogo et al., 2002). The replication stress-inducing agent HU causes an accumulation of ssDNA foci in cells in S-phase cells but not outside S-phase (Figure 1C) (Sogo et al., 2002). In addition to IR and replication stress, ssDNA accumulation can be observed with other agents of interest targeting the DDR (Kilgas et al., 2021; He et al., 2018; Kijas et al., 2015; Broderick et al., 2016; Jachimowicz et al., 2019; Bai et al., 2019). In addition, not only the presence of DNA damaging agents, but also the dose and duration of treatment as well as the recovery times will affect the intensity and/or number of BrdU foci being generated (Zhou and Paull, 2021; Despras et al., 2010).

## Quantification and statistical analysis

The number of BrdU foci (ssDNA foci) is determined by Fiji/Image J with a semi-quantitative approach:

1. Open original microscopy images (the CZI file format for the Zeiss 710 confocal microscope used here) by drag and drop option in Fiji (Figure 2A).

**Figure 2:**
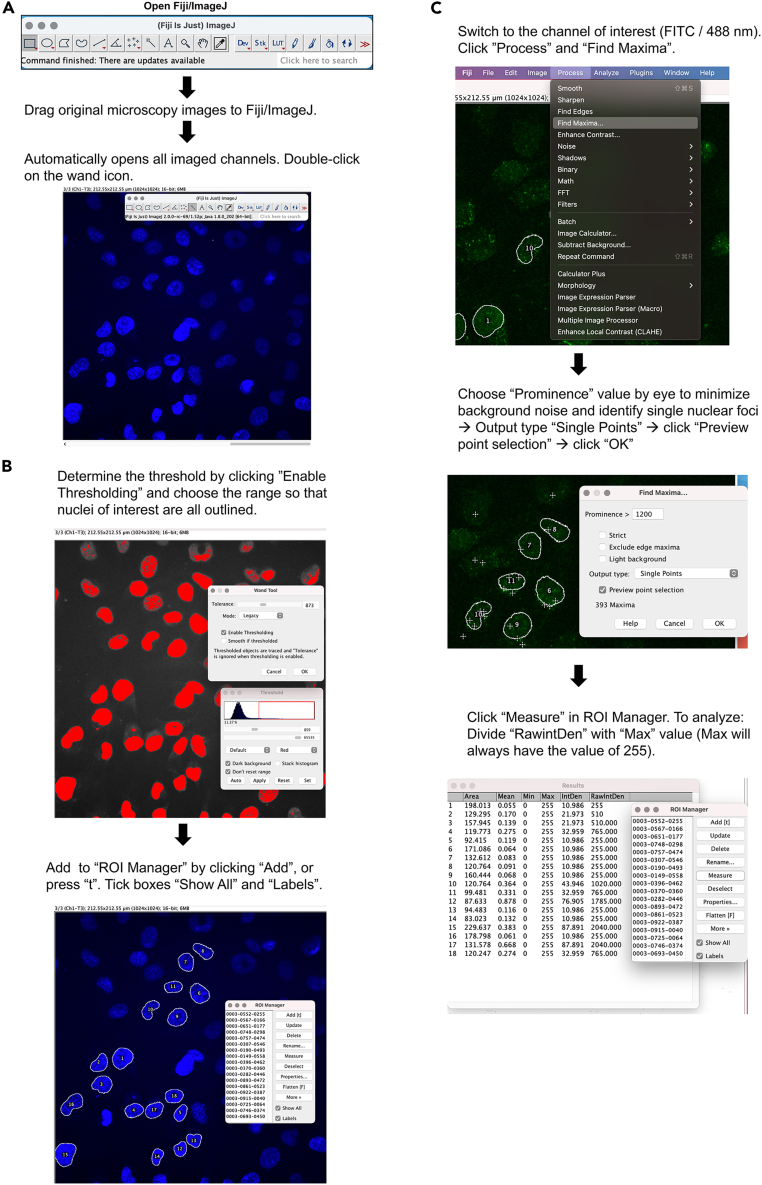
Individual steps for semi-automated assessment of BrdU/ssDNA foci Steps and representative images that illustrate BrdU foci quantification using Fiji/ImageJ software (A) Steps showing how to open original microscopy images in Fiji program. (B) Steps showing how to choose individual nuclei in DAPI channel and add the chosen cells to the ROI Manager. (C) Steps showing how to set tolerance levels to recognize individual foci and subsequently calculate the total number of BrdU foci in cells.
2. Select cells in the DAPI channel and click on the “wand tool”, set tolerance and enable thresholding to select individual nuclei. These can then be added to “Region of interest (ROI) Manager” by clicking “Add” or pressing “t” (Figure 2B).
3. Move to the channel of interest to count individual foci. For this example, it is the FITC channel (Ex 488 nm) for BrdU foci. Select process ▸ find maxima ▸ output type: “single points” ▸ choose “Prominence” (threshold levels of foci recognition) until individual single points are identified with minimal background noise (Figure 2C).

**CRITICAL:** Keep the same threshold levels across all conditions during analysis. Do not change image contrast or intensity (see troubleshooting 6).

4. When nuclei are selected on an image, click “Find maxima” and then “Measure” from the ROI Manager.
5. Number of foci can be determined by dividing the raw intensity (“RawIntDen”) value with the “Max” value.

All statistical analysis was done using GraphPad software (PRISM 8). A value of p ≤ 0.05 was considered statistically significant.

## Limitations

The major limitation of this protocol is that it is a semi-quantitative way of analyzing the amount ssDNA foci generated. Despite being a useful way to easily and reliably estimate the number and/or brightness of ssDNA foci generated, when a more quantitative and precise method is required that measures the extent of resection from specific DSB sites, quantitative PCR (qPCR)-based assays might be the best option (Zhou and Paull, 2021). However, it is worth noting that qPCR-based methods do require the generation of stable cell lines, which is more labor-intensive than the current protocol. Additionally, other ways to quantitatively measure DNA end resection are in-gel assays with fluorophores/radioactive isotopes (Bantele et al., 2019), FACS-based methods to quantify ssDNA (Forment and Jackson, 2015) and the DNA fiber-based Single Molecule Analysis of Resection Tracks (SMART) assay. The SMART assay resembles the current protocol, but instead of analyzing BrdU foci, it is based on anti-BrdU antibody detection on DNA fibers, providing a quantitative readout of the length of DNA resection tracks in different conditions (He et al., 2018; Altieri et al., 2020). Finally, image acquisition and analysis using Fiji/Image J are relatively time-consuming and labor-intensive but depend on the number of samples being analyzed.

## Troubleshooting

### Problem 1: Insufficient fixation before BrdU staining

When fixation time or the concentration of the fixative is not sufficient, the signal-to-noise ratio for BrdU will decrease, potentially compromising the quality of analysis. This will also lead to loss of sample.

### Potential solution

Perform further fixation prior to staining. Altering the fixative (changing PFA to ice-cold methanol) or increasing fixation time could help. Please refer to protocol step 17 for further information.

### Problem 2: Cells are over fixed

When fixation time or the concentration of the fixative is too high, antibody-binding efficiency will decrease due to over-crosslinking. Longer fixation times with PFA will also increase autofluorescence, hampering analysis of the samples.

### Potential solution

Reduce the duration of fixation, or alternatively use a lower concentration of PFA. Please refer to protocol step 17 for further information.

### Problem 3: Cells were not properly permeabilized and cell detachment was observed

When cells are not properly permeabilized, antibodies will not be able to access the intracellular components and there will be no discernible BrdU foci or nuclear staining of BrdU. Also, when the pre-extraction buffer is left on for longer than indicated or performed at 20°C–22°C, this will lead to loss of cells.

### Potential solution

Fixing cells with methanol will permeabilize cells, so there is no need to add Triton X-100 in extraction buffers, which potentially can avoid problems around cell detachment. However, we recommend the use of PFA for cell fixation for this assay as PFA works better for signal clarity as well as preserving cell structure compared to methanol fixation in our protocol. When cells are fixed with PFA, cells need to be permeabilized with Triton X-100, which is included in the pre-extraction buffer recipes outlined above (see Materials and Equipment). Alternatively, if cell detachment is a persistent issue, the concentration of Triton X-100 could be lowered from 0.5% to 0.25%. Also, handle all coverslips carefully to avoid cell detachment after pre-extraction: keep coverslips on ice, add PBS gently to 12-wells and not directly onto the coverslip. Also, avoid scratching the top of the coverslip with forceps when moving samples around. Refer to protocol step 15 for further information

### Problem 4: Not enough primary antibody

When primary antibody concentration or incubation time is too low, then not enough primary antibody can bind the protein of interest, causing faint samples and low signal-to-noise ratio.

### Potential solution

Using a higher concentration of the primary antibody could eliminate this problem. Additionally, incubating for longer, such as 4°C overnight (approximately 16 h) in the dark, could result in better signal. Please refer to protocol step 26 for further information.

### Problem 5: High background

High background fluorescence can result for various reasons: low signal-to-noise ratio from cross-reactivity between different fluorescent channels; primary antibody is not specific enough or not at the right concentration; not enough blocking or inappropriate blocking reagent; not enough washing in between staining steps; over and under fixation of cells; excessive drying of coverslips prior to mounting.

### Potential solution

Use of specific antibodies at the recommended concentrations; using siRNA-mediated depletion of protein of interest to see loss of signal compared to siRNA control; checking any fluorescence in an unstained cell. If autofluorescence is present despite siRNA-mediated knockdown (KD) or in unstained control, reduce the concentration of primary and/or secondary antibody, increase duration of blocking time, or consider changing the blocking reagent. If imaging more than one fluorescent probe, choose excitation and emission spectra that do not overlap between different antibodies. Furthermore, dry coverslips very briefly before mounting and do not let the coverslips dry out during antibody incubation or washing times. Finally, make sure to wash coverslips at least three times and for 5 min each in between staining steps and prior to mounting. Please refer to protocol step 41 for further information.

### Problem 6: Incorrect intensity settings at the image acquisition step

**Figure 3:**
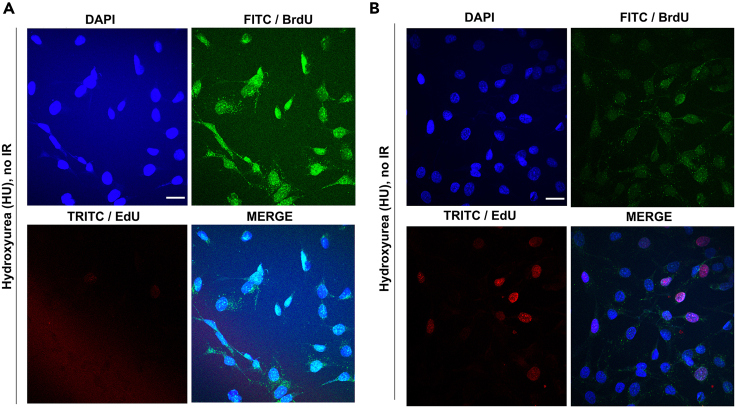
Representative confocal microscopy images illustrating both overexposed and correctly exposed images (A) T24 cells were treated with 500 μM HU for 1 h and stained with DAPI, BrdU antibody (FITC/green channel) and fluorescent dye (“Click-iT” Alexa Fluor 555) to mark S-phase cells (TRITC/red channel). All three channels show representative images of incorrect intensity, where images were taking when signal was too saturated with high background. Also note the high background cytoplasmic foci becoming visible in the green/BrdU foci channel (see also troubleshooting 3). (B) Same conditions as in (A), but correctly exposed DAPI, green and red channels. Scale bar = 30 μm. See troubleshooting 6 for further details.

Incorrect intensity can lead to either signal being too low to visualize foci or signal being saturated with high background (Figures 3A and B).

### Potential solution

Before acquiring images, test out a few controls and a few treated conditions and set the intensity at the right level. Signal should not be saturated even in the brightest condition, and signal should have just enough brightness so foci will be clearly visible without background noise. Please refer to protocol step 45 for further information. Please refer to protocol step 3 of “quantification and statistical analysis”.

### Problem 7: Fluorescence bleaching

When the laser is left on the sample for too long, sample bleaching and subsequent loss of signal will occur.

### Potential solution

Avoid overexposing samples to the laser by switching off the light source as soon as imaging is done. Also, store all your coverslips in the dark and avoid any light exposure in between image acquisition and sample storage. Please refer to protocol step 45 for further information.

## Resource availability

### Lead contact

Further information and requests for resources and reagents should be directed to and will be fulfilled by the lead contact, Kristijan Ramadan (kristijan.ramadan@oncology.ox.ac.uk).

### Materials availability

This study did not generate new unique reagents.

## Data Availability

This study did not generate any unique datasets or code.
